# Distance is “a big problem”: a geographic analysis of reported and modelled proximity to maternal health services in Ghana

**DOI:** 10.1186/s12884-022-04998-0

**Published:** 2022-08-31

**Authors:** Winfred Dotse-Gborgbortsi, Kristine Nilsen, Anthony Ofosu, Zoë Matthews, Natalia Tejedor-Garavito, Jim Wright, Andrew J. Tatem

**Affiliations:** 1 School of Geography and Environmental Science, University of Southampton, Southampton, S017 1BJ UK; 2 WorldPop, School of Geography and Environmental Science, University of Southampton, Southampton, UK; 3 Department of Social Statistics and Demography, University of Southampton, Southampton, UK; 4 Ghana Health Service, Headquarters, Accra, Ghana

**Keywords:** Maternal health services, Accessibility of health services, Geographic information systems, Spatial analysis, Childbirth, Healthcare disparities

## Abstract

**Background:**

Geographic barriers to healthcare are associated with adverse maternal health outcomes. Modelling travel times using georeferenced data is becoming common in quantifying physical access. Multiple Demographic and Health Surveys ask women about distance-related problems accessing healthcare, but responses have not been evaluated against modelled travel times. This cross-sectional study aims to compare reported and modelled distance by socio-demographic characteristics and evaluate their relationship with skilled birth attendance. Also, we assess the socio-demographic factors associated with self-reported distance problems in accessing healthcare.

**Methods:**

Distance problems and socio-demographic characteristics reported by 2210 women via the 2017 Ghana Maternal Health Survey were included in analysis. Geospatial methods were used to model travel time to the nearest health facility using roads, rivers, land cover, travel speeds, cluster locations and health facility locations. Logistic regressions were used to predict skilled birth attendance and self-reported distance problems.

**Results:**

Women reporting distance challenges accessing healthcare had significantly longer travel times to the nearest health facility. Poverty significantly increased the odds of reporting challenges with distance. In contrast, living in urban areas and being registered with health insurance reduced the odds of reporting distance challenges. Women with a skilled attendant at birth, four or more skilled antenatal appointments and timely skilled postnatal care had shorter travel times to the nearest health facility. Generally, less educated, poor, rural women registered with health insurance had longer travel times to their nearest health facility. After adjusting for socio-demographic characteristics, the following factors increased the odds of skilled birth attendance: wealth, health insurance, higher education, living in urban areas, and completing four or more antenatal care appointments.

**Conclusion:**

Studies relying on modelled travel times to nearest facility should recognise the differential impact of geographic access to healthcare on poor rural women. Physical access to maternal health care should be scaled up in rural areas and utilisation increased by improving livelihoods.

**Supplementary Information:**

The online version contains supplementary material available at 10.1186/s12884-022-04998-0.

## Introduction

Maternal mortality has declined globally, but progress in reducing pregnancy and childbirth-related deaths in Sub Saharan Africa is slow [1]. Maternal deaths have reduced by 38% within approximately two decades from the year 2000, but the gradual annual rate of change (2.9%) is not sufficiently rapid to achieve the Sustainable Development Goal for reducing maternal mortality by 2030 [2]. Most maternal deaths (94%) happen in poorly resourced settings [3], and Sub Saharan Africa has the highest estimated mortality, which is declining only gradually. Like its neighbours, Ghana has a high maternal death rate, declining by only 2.7% every year. The distribution of maternal deaths by location and socio-demographic group can be uneven [4].

The leading causes of maternal mortality are severe bleeding after childbirth, pregnancy-induced high blood pressure and infections after childbirth [3, 5]. These causes are responsible for three-quarters of maternal deaths. Therefore, the primary interventions to prevent maternal deaths are quality emergency obstetric care provided by skilled health professionals during pregnancy, childbirth and the first 2 days after birth [6].

Pregnancy and childbirth-related complications can be identified and managed when women receive timely quality health services, but they have to overcome geographic and transport barriers to access care [7]. Even among populations with higher socio-economic status, health services might not be available or accessible [8], leading to complications and death. In several locations across Africa, maternal health services are unavailable, inaccessible, and often below the recommended geographic accessibility requirements [9].

Proximity to quality health services is a crucial determinant of maternal mortality, stillbirths, and child mortality because children and women living closer to health facilities have better health outcomes [10–12]. Women living farther from health facilities might either not use them [13] or reach the health facility late with a life-threatening complication. Cost of transportation, lack of public transport, and poor road networks are other journey-related factors that could hinder women’s ability to reach a health facility [14].

Geographic accessibility is one of the main barriers that inhibit women from using maternal health services in Africa [15]. Many Sub-Saharan Africa countries have at least one sub-national area lacking at least one hospital per half a million population [9]. About half of Ghanaian women live two or more hours’ drive from health facilities providing comprehensive obstetric care [16]. Thus, it is unsurprising that some women in Ghana describe the distance they travel to access health services as a “big problem” [17].

Previous studies have compared self-reported geographic healthcare access measures and modelled travel times to nearest health facility [18–21]. Different approaches were used to measure self-reported distance and modelled travel times. Self-reported healthcare proximity was deduced from journey start/end times [18, 20] or distance reported by patients or their relatives [19–21]. The modelled travel times were based on road networks or impedance grid cells estimating the cost of travel. Generally, these studies have found that modelled travel times are lower than self-reported travel times [19–21]. These comparisons have often been based on self-reported measures of geographic access available only in one country or sub-region via a research study, rather than on a self-reported geographic access measure incorporated into an international household survey series.

As part of the Demographic and Health Surveys (DHS) implemented in several countries, women are asked whether the distance to the health facility is a big problem when visiting a health facility with illness [22]. Some studies have relied on this DHS question to assess the uptake of maternal health services. Their results found self-reported challenges with distance were associated with lower use of antenatal, skilled birth and postnatal services, but none compared self-reported and modelled distance [23–25]. Furthermore, studies comparing self-reported versus modelled travel times [18–21] have not evaluated the standard DHS question by comparing self-reported distance challenges to modelled travel times. Therefore, there is currently no evidence for the robustness of the DHS question concerning distance-related challenges accessing healthcare.

This paper firstly aims to assess the relative effects of modelled travel times and self-reported problems with distance to healthcare on skilled attendance at birth. Secondly, it aims to assess socio-demographic factors associated with women reporting problems with distance to healthcare.

## Methods

### Data

The 2017 Ghana Maternal Health Survey (GMHS), a special DHS, sampled 25,062 women aged 15 to 49 years from 900 enumeration areas representative at national and regional levels [17]. The GMHS used a two staged cluster sample design with rural/urban stratification to collect data about women’s experiences and use of maternal health services between June and October 2017, achieving a 99% response rate. The GMHS also recorded the geographic location of clusters. This study includes information on women aged 15–49 who were asked about their last birth in the 5 years before the 2017 GMHS survey. Ghana Health Service (GHS) data on the location of health facilities providing birthing services in 2017 and spatial topographic data were used to model travel times. Spatial data representing terrain, land cover, roads, rivers/water bodies and topography were included to model the travel time to health facilities [26–28].

### Modelling travel times

Travel time to the nearest health facilities providing birthing services was modelled in Accessmod version 5 [29], a free tool for measuring access to health services. To measure the travel time to each health facility, we first created an impedance cost surface (a gridded layer representing the difficulty of travel) by combining landcover, terrain, roads, and water bodies (e.g. rivers and lakes). Where roads of different classes met, the road with the maximum speed was prioritised. We assumed that patients would walk on all land cover types, then use mechanised transport on roads. Therefore, walking speeds were 5 kmh^− 1^ or less in forests, woodlands and croplands and other landcover types depending on how easily they can be traversed [30]. Following traffic regulations in Ghana, primary, secondary and tertiary roads were assigned 90 kmh^− 1^, 50 kmh^− 1^ and 30 kmh^-1,^ respectively. The model ensures that the terrain’s steepness affects travel speed towards a health facility for persons walking or bicycling.

Via the impedance surface, we estimated the travel times from each GMHS cluster location to the nearest health facility where birthing services are provided. Birth counts from routine GHS health data indicate health facilities providing birthing services. The longitudes and latitudes for DHS clusters are intentionally randomly displaced within two kilometres in urban clusters and five or up to ten in rural areas for data protection reasons. Therefore, we used two and five-kilometre buffers to compute the median travel times around urban and rural clusters respectively, calculating medians because travel times had skewed distributions within these buffers. These recommended distance buffers mitigate the likely effects of cluster displacement [31].

### Statistical analysis

#### Study variables

The DHS asks respondents if distance is a big problem in accessing healthcare when sick. The binary response from this question was the main outcome studied. The DHS questionnaire asked women the following question: “Many different factors can prevent women from getting medical advice or treatment for themselves. When you are sick and want to get medical advice or treatment, is each of the following a big problem or not a big problem: The distance to the health facility?” Response options: 1. Big problem 2. Not a big problem [32].

Secondly, we assessed the effect of proximity and socio-demographic variables on skilled birth attendance (SBA). For SBA, a woman was assisted by a skilled attendant if the most qualified person during childbirth was a midwife, doctor or nurse.

The predictor variables were the number of antenatal care (ANC) appointments and postnatal care (PNC) within 48 hours after birth. Other variables included were age, rural-urban, wealth, region, health insurance and education [33].

In this study, ANC and PNC were defined as skilled if the service provider was a midwife, doctor or nurse. When two or more providers are present, the highest qualified service provider is used to classify skilled ANC and PNC. Based on the older WHO recommendation, the number of skilled ANC appointments were recoded into three (no ANC appointments, one to three, four or more) [34]. Similarly, timely PNC was defined as any woman who received a health check or visits from a skilled provider within 48 hours after delivery, while in the health facility or at home following delivery.

The women’s ages were grouped into three classes (15 to 20 years, 21 to 30 years, 31 to 49 years). Household wealth quintiles were collapsed into three classes (poor, middle, and rich). We combined the “poorest” and “second” into “poor” and “fourth” plus “wealthiest” as “rich”. The DHS used household assets, livestock, drinking water source, type of toilet, type of cooking fuel, and building structure to construct the wealth index via a principal component analysis [35]. The highest education attained were recoded as no formal education, primary, secondary, and higher education. Due to the high proportion of missing values for women covered by health insurance, registration with a health insurance scheme was used as a proxy for insurance cover.

#### Descriptive analysis

The estimated median travel time between groups was reported with inter-quartile range (IQR). For categories with two levels, the differences in travel time were tested with the Wilcoxon rank sum test, whereas the Kruskal Wallis test was applied to groups with three or more levels. Non-parametric tests were chosen because the travel time distribution was skewed. To test for association between the reported distance and the independent variables, chi square tests were used. The descriptive statistics were presented in tables and plots to visualise the difference between groups.

#### Modelling the relationship between proximity, socio-demographic characteristics and skilled birth attendance

Logistic regression models were used to estimate the relationship between reported distance problems and SBA, controlling for socio-demographic and maternal health characteristics. The skilled birth outcome was chosen because it is the key determinant of maternal health outcomes [7]. Crude odds ratios were estimated between skilled birth attendance and each independent variable. All independent variables in the crude model associated with SBA at 10 % significance level were added to the adjusted models to allow for associations that can be insignificant in the crude model but change in the presence of other variables [36]. We tested all other associations at 5 % significance.

#### Modelling the relationship between travel time, socio-demographic characteristics and self-reported distance

Furthermore, a logistic regression analysis was conducted to estimate the relationship between socio-demographic backgrounds and reported challenges with distance. The outcome variable in this model was the binary self-reported distance. The independent variables were the socio-demographic variables and modelled travel time. Multicollinearity for both models was checked with a variance inflation factor threshold set at ten. We used the likelihood ratio test to compare the models. Finally, we included survey weights to correct for sampling and non-response error.

## Results

### Socio-demographic characteristics and reported distance compared

The analysis included 2090 women (2090 weighted, 2210 unweighted) of which 89% attended four or more ANC appointments, 86% had SBA, and 81% received skilled PNC promptly. All three maternal health services (ANC, SBA, PNC) were significantly associated with reported distance problems (Table 1). Almost one third (28%) of respondents reported distance as a big problem in accessing healthcare when sick. Most women receiving the recommended maternal health services did not find distance challenging. Majority (70.2%) of women reporting distance challenges were in rural areas. Almost half (46.1%) of women who did not find distance challenging were wealthier. Furthermore, 8.8% of women who reported no distance difficulties had no formal education compared to double the proportion (16.3%) among women with challenges. Younger women found distance more challenging than older ones. Most women were registered for health insurance and more women without health insurance were worried about distance than women with health insurance. All socio-demographic variables were significantly associated with self-reported distance challenges.

**Table 1:** Weighted frequencies of self-reported distance by socio-demographic characteristics and maternal health outcomes (weighted *n* = 2090)

Characteristic	Is distance a big problem?		Chi square *p* value	Total (%)
	Yes (%)	No (%)		
**Maternal health outcomes**				
No ANC appointment	7 (1.4)	21 (1.3)	< 0.001	28 (1.3)
1 to 3 appointments	75 (14.6)	125 (7.9)		200 (9.6)
Four or more appointments	433 (84.1)	1428 (90.7)		1861 (89.1)
No SBA	107 (20.7)	188 (11.9)	< 0.001	295 (14.1)
SBA	409 (79.3)	1386 (88.1)		1795 (85.9)
No PNC	124 (24.1)	274 (17.4)	< 0.001	398 (19.1)
PNC	391 (75.9)	1300 (82.6)		1691 (80.9)
**Rural/urban**				
Rural	362 (70.2)	652 (41.4)	< 0.001	1014 (48.5)
Urban	154 (29.8)	923 (58.6)		1077 (51.5)
**Region**				
Ashanti	54 (10.5)	271 (17.2)	< 0.001	325 (15.6)
Brong Ahafo	28 (5.4)	176 (11.2)		204 (9.8)
Central	43 (8.4)	149 (9.5)		192 (9.2)
Eastern	60 (11.7)	169 (10.7)		229 (11.0)
Greater Accra	56 (10.9)	325 (20.6)		381 (18.2)
Northern	90 (17.5)	102 (6.5)		192 (19.2)
Upper East	32 (6.2)	63 (4.0)		95 (4.5)
Upper West	19 (3.7)	36 (2.3)		55 (2.6)
Volta	68 (13.2)	106 (6.7)		174 (8.3)
Western	64 (12.5)	179 (11.4)		243 (11.6)
**Wealth**				
Poor	327 (63.4)	502 (31.9)	< 0.001	1136 (51.4)
Middle	82 (15.9)	346 (22.0)		428 (20.5)
Rich	107 (20.7)	726 (46.1)		833 (39.9)
**Age group**				
15–20	205 (39.8)	467 (29.7)	< 0.001	672 (32.2)
21–30	277 (53.8)	977 (62.1)		1254 (60.0)
31–49	33 (6.4)	130 (8.3)		163 (7.8)
**Health insurance**				
Registered with health insurance	434 (84.3)	1412 (89.7)	0.001	1846 (88.4)
Not registered with health insurance	81 (15.7)	162 (10.3)		243 (11.6)
**Education**				
No formal education	84 (16.3)	139 (8.8)	< 0.001	223 (10.7)
Primary	316 (61.4)	888 (56.4)		1204 (57.6)
Secondary or higher	115 (22.3)	548 (34.8)		663 (31.7)

### Modelled travel times, reported distance challenges, maternal health services and socio-demographic characteristics compared

**Fig. 1:**
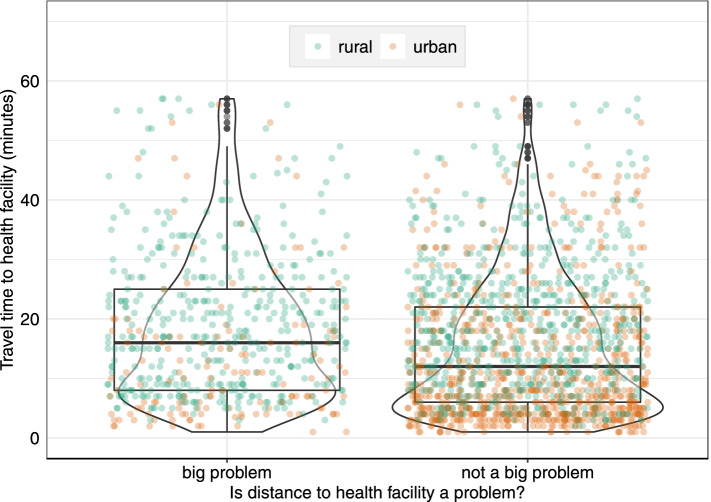
Modelled travel time to the nearest health facility providing birthing services versus women reporting distance as a big problem for rural and urban GMHS cluster locations. The boxplot shows the median travel times and interquartile range, the dots present the rural/urban distribution of the women and the violin plots show the density distribution of the women. To improve comparison and visualisation, 113 women from 32 clusters with travel time beyond 60 min were excluded from the plot. Unfiltered plot is in Supplemental Appendix (Fig. S1)

The median travel time to the nearest health facility for women reporting challenges with distance was 17 min (IQR 21), significantly longer (*p* < 0.001) than that for women not reporting such challenges (13 min; IQR 17). Among women who did not have problems with distance, most urban women were below the median travel time (Fig. 1).

**Fig. 2:**
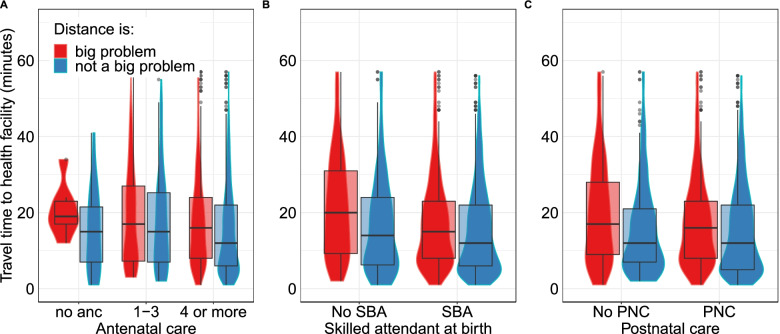
Modelled travel time to the nearest health facility providing birthing services versus women reporting distance as a big problem for utilisation of **A**. Antenatal, **B**. Skilled birth and **C**. Postnatal care services. The boxplot shows the median travel times and interquartile range and the violin plots show the density distribution of the women. To improve comparison and visualisation, 113 women from 32 clusters with travel time beyond 60 min were excluded from the plot. Unfiltered plot is in Supplemental Appendix (Fig. S2)

Women who reported distance as a problem and did not attend ANC lived further from a health facility than women attending four or more ANC appointments (Fig. 2). The pattern among ANC users was similar to SBA and PNC. Generally, women who lived closest to health facilities received the recommended maternal health services compared to more distant ones. More women lived at shorter travel times to health facilities, and fewer at longer travel times, as seen in the violin plots in Fig. 2.

**Fig. 3:**
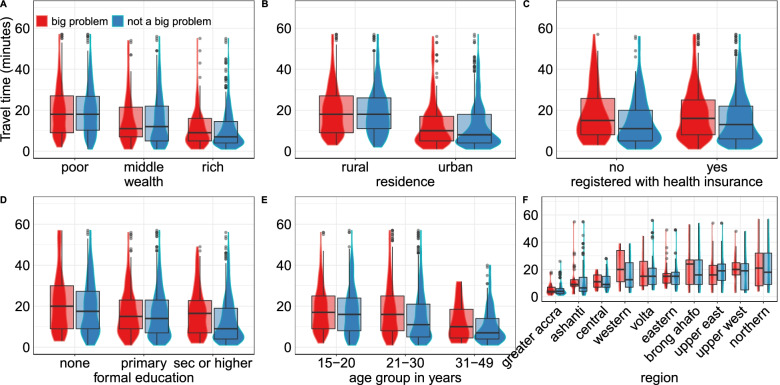
Travel time to the nearest health facilities providing birthing services versus reported distance as a big problem compared by **A** Wealth, **B** Residence **C** Health insurance, **D** Education, **E** Age group, and **F** Region. The boxplot shows the median travel times and interquartile range and the violin plots show the density distribution of the women. To improve comparison and visualisation, 113 women from 32 clusters with travel time beyond 60 min were excluded from the plot. Unfiltered plot is in Supplemental Appendix (Fig. S3)

The travel times decreased among wealthier households (Fig. 3). Furthermore, rich and middle wealth households reporting distance problems were closer to health facilities than poor households with no distance challenges. In poor and rural households, the travel times for reported distance groups were similar. Interestingly, women who did not find distance challenging in rural locations had higher travel times than urban-dwelling women who reported challenges with distance. Travel times decreased with an increase in education, and the most substantial difference was among women with secondary education. The difference between the groups in Greater Accra, Eastern and Volta regions was marginal. In contrast, Western and Brong Ahafo regions had the most considerable difference in travel time for reported distance. The Upper West was the only region where women who did not find distance challenging had longer travel times near their cluster.

Table 2 shows the travel times for levels within maternal health and socio-demographic variables. There was a statistically significant difference within the levels for ANC, SBA, PNC, rurality, region, wealth, age groups, and education. Health insurance registration was the only variable with no difference as women with no health insurance travelled 15 min, 1 min more than those who were registered. The largest gaps between groups were in rural/urban and wealth. Women in rural areas travelled 11 min more than women in urban areas, while women in rich households journey 12 min less than poor women. Greater Accra and Ashanti regions have the shortest travel times, below 10 min.

**Table 2:** Modelled travel times to nearest health facility for levels within socio-demographic and maternal health service variables compared

Variable	Median travel time in minutes (IQR)	Test for difference in travel time*
**Maternal health outcomes**		
No ANC	19 (20)	0.009
1–3	17 (20)	
> 4	14 (17)	
No SBA	18 (23)	< 0.001
SBA	14 (17)	
No PNC	17 (23)	< 0.001
PNC	14 (17)	
**Rural/urban**		
Rural	19 (18)	< 0.001
Urban	8 (14)	
**Region**		
Ashanti	7 (10)	< 0.001
Brong Ahafo	16 (19)	
Central	11 (10)	
Eastern	15 (8)	
Greater Accra	4 (3)	
Northern	23 (32)	
Upper East	18 (12)	
Upper West	20 (18)	
Volta	15 (14)	
Western	13 (21)	
**Wealth**		
Poor	20 (20)	< 0.001
Middle	12 (18)	
Rich	8 (11)	
**Age groups**		
15–20	17 (18)	< 0.001
21–30	13 (18)	
31–49	8 (13)	
**Health insurance**		
Registered with health insurance	14 (19)	0.848
Not registered with health insurance	15 (18)	
**Education**		
No formal education	21 (26)	< 0.001
Primary	15 (17)	
Secondary or higher	11 (16)	

*P-values are from non-parametric tests; Wilcoxon test for binary groups and Kruskal Wallis test for groups with two or more levels

### Relationship between distance, socio-demographic characteristics and skilled birth attendance

**Fig. 4:**
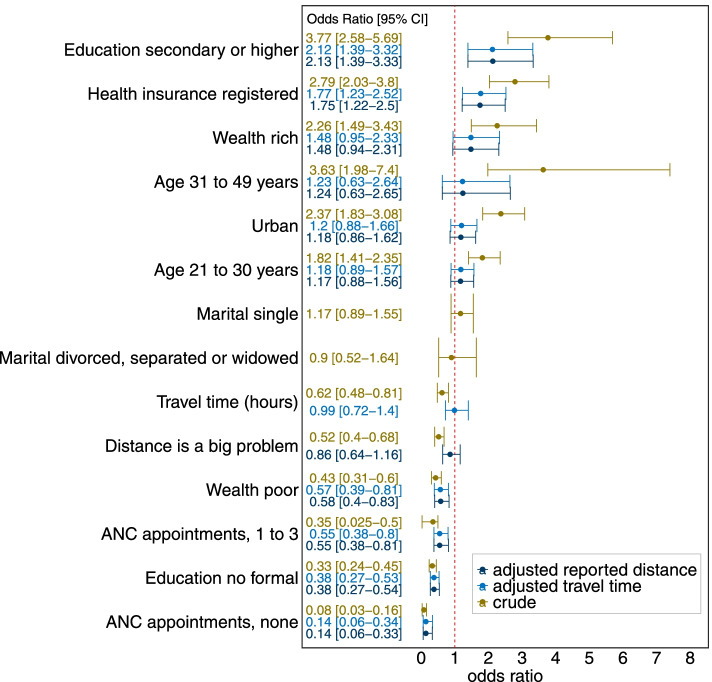
Crude and adjusted relationship between reporting distance as a problem and skilled birth attendance. Statistically significant associations do not cross the red line

The logistic model results predicting SBA are presented in Fig. 4. The crude odds ratios showed wealth, urban, health insurance, higher education and older age increased the likelihood of using SBA. Higher education had the highest crude association with SBA. Women with secondary or tertiary education are 3.8 times more likely to have SBA than women with primary education. In contrast, women who did not attend ANC were 92% less likely to have SBA than those with four or more appointments. Self-reported distance had a stronger crude association with SBA than modelled travel time. Women reporting distance challenges had 48% reduced odds of SBA while each additional hour to the nearest health facility reduced the odds by 38%. Poor households and no formal education were associated with lower SBA utilisation.

After adjusting for socio-demographic and maternal health characteristics, only health insurance and higher education were significantly associated with increased SBA uptake. All the variables that reduced the likelihood of SBA in the crude model (being poor, lacking formal education, no ANC appointments, one to three ANC appointments) remained statistically significant. Self-reported distance and modelled travel times were no longer significant in the adjusted models.

A likelihood ratio test suggests that a base model without any of the distance variables is statistically different from the null model (chi square: 228, *p* < 0.001). The base model included ANC, rural/urban, health insurance, wealth, education and age. Then, reported distance and modelled travel time were added to a base model to investigate their relative association with SBA. The analysis showed self-reported distance (chi square: 0.3910, *p* < 0.53) and travel time (chi square: 0.0224, *p* < 0.881) did not significantly change the base model.

### Factors associated with reported challenges with distance

**Fig. 5:**
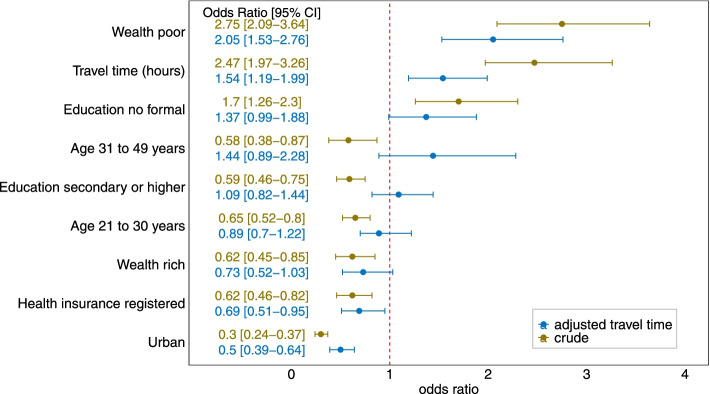
The relationship between socio-demographic characteristics, modelled travel times and self-reported distance challenges

The relationship between socio-demographic characteristics and modelled travel time on reported challenges with distance are shown in Fig. 5. In the crude model, socio-demographic characteristics and modelled travel time were significant predictors of self-reported distance. In the adjusted model, poverty, living in rural areas and an hour’s increase in modelled travel time to the nearest health facility significantly increased the odds of a woman reporting distance challenges. Though not significant in the adjusted model, women without formal education had 37% higher odds of reporting challenges with distance than primary educated women. Women registered with health insurance had 41% decreased odds of reporting challenges with distance.

## Discussion

This is the first study comparing modelled travel times with distance challenges reported by women via the DHS survey questions. Our comparison found that women reporting distance as a challenge in accessing healthcare had a significantly longer modelled travel time to the nearest health facility. Generally, women with poor socio-demographic backgrounds had longer travel times to their nearest health facility. The travel times varied by region and urban areas had shorter journey times. Women with better maternal health outcomes (skilled birth attendant, four or more skilled ANC appointments and timely skilled PNC) had shorter travel times to the nearest health facility. There is thus consistency between modelled travel times and women’s responses to the DHS question on distance challenges accessing healthcare, suggesting the former is a valid construct reflecting geographic healthcare access.

Our findings support the experience of women who said distance to the health facility is challenging when they are sick. These women are likely to be seeking ANC, SBA, PNC and other maternal health services at more distant health facilities than other women. This study and several others found associations between healthcare proximity and maternal health service uptake [25, 37]. For instance, a woman attributed her stillbirth to her community’s lack of a health facility, leading to her travelling further to give birth in a nearby region [38]. Lack of a nearby health facility at reasonable travel times can lead to home births and adverse birth outcomes.

We found modelled travel times underestimate geographic access problems for poor, rural and less educated women. The underestimation of geographic access problems among disadvantaged women is consistent with similar studies [18]. However, our travel times could be lower than the real journey times for these groups of women due to our modelling approach [21]. Poor, rural women may be more likely to experience delays waiting for infrequent public transport such as buses, and public transport may be slower than other forms of mechanised transport. Such delays are not incorporated into modelled travel times. Finally, our modelled travel time assumes a constant travel speed on each road class and does not account for road conditions that might affect the journey time, particularly in rural areas. Incorporating road conditions in travel time models could improve modelled travel time estimates.

Geographical barriers limit access to healthcare and slow universal healthcare progress [39]. Geographic barriers can include the quality of roads, cost of transportation, and the travel time to a nearby health facility where services are available, ready and fit for purpose. To reach health facilities, women must overcome contextually varied geographic barriers [14]. For example, women in rural areas might travel at least twice as far as their urban counterparts because there are likely fewer health facilities, poorer roads, and less equipped health facilities in rural areas. Although health facilities can be closer to urban dwellers, the urban poor might be disadvantaged than the rural poor because of expensive services in nearby private facilities [40]. Comparatively, urban women might live closer to health facilities but have longer travel times due to traffic congestion [41]. In some settings, the needed intervention might be better transportation services in the form of links to major roads, asphalted surfaces, availability of vehicles and affordable cost of travel.

Both geographic access measures are related to SBA in unadjusted models but not following adjustment for other characteristic such as rurality, poverty and education. Maternal and household characteristics can moderate the relationship between distance and the utilisation of services [42]. Health care access transcends geographic access to include other factors such as the cost of services, social access, the quality of care, acceptability amongst other dimensions of access [43, 44]. In Ghana, the cost of services and decision-making autonomy were key determinants of access to health services [45]. Also, maternal literacy, health insurance and household wealth were linked with women’s health seeking autonomy and SBA utilisation [45, 46]. Rural women are predisposed to unfavourable maternal health outcomes than urban women because they are mostly poor, less educated and live farther from health facilities. For instance, poorer women might not afford services; less educated women might be poorer and have lower obstetric risk perceptions. Therefore, addressing the social determinants of health could improve socioeconomic status and might promote better maternal health outcomes [47].

The self-reported distance problems in the DHS correlated with the estimated travel time to the nearest health facility. The results underscore the relevance of perceived challenges or subjective ratings in the DHS. Responses to the DHS survey question on proximity to healthcare services are broadly consistent with modelled travel times, suggesting this construct is valid. Also, women from a cluster location receiving care at the same health facility might rate the distance differently. For instance, Fig. 3A shows that wealthier households reporting distance as a problem lived closer to health facilities than poor households with no distance challenges. A plausible explanation could be self-reported distance reflecting mode of transportation and other difficulties in accessing care than modelled travel time. For example, urban women might reach longer distances quicker in a mechanised vehicle than urban women travelling shorter distances on foot. Therefore, the question should be maintained and possibly expanded to include perceived quality of maternal health care. Were a service quality question to be included in the DHS, it could be evaluated through spatial linkage to service provision assessments. If patient-perceived quality of maternal health care correlates with service provision assessment quality indices, they can inform spatially targeted healthcare quality improvements.

This study combines different data sources to evaluate proximity to maternal health services. We joined routine health data, DHS, and travel time models to assess proximity to health services, similar to other studies integrating spatial data [48]. Each data source has its relevance. First, routine data shows where services exist by examining the counts of service users captured in health management information systems. Then, service provision assessments measure the quality of the services.

Furthermore, household or neighbourhood wealth characteristics can be derived from census, surveys or remote sensing [49]. Finally, proximity measures can be estimated using spatial models implemented in this study. These data sources present opportunities to explore and unpack spatial inequalities among rural-urban, urban poor and other sub-groups. Through such integrated analysis, areas with poor access to care will be apparent for locating new facilities or upgrading poor ones.

The current results are specific to Ghana and could change when new health facilities are built, or road conditions improve before subsequent surveys. However, the analysis can be expanded to include other countries in Sub-Saharan Africa because the DHS survey is widely available. Also, the analysis could be conducted using multiple DHS waves to assess changes in reported distance problems. Global travel time to health facility data [50] is essential for large scale sub-regional analysis comparing many countries. However, travel time to all health facilities can only be used as a proxy for access to specific health services because health facilities can be distinct in the services they provide. Therefore, for contextual analysis investigating access to maternal health services, studies can rely on routine health data to identify locations where services are available to estimate journey times.

We used the most recent survey data available and relied on known health facility locations to measure travel times. Also, we relied on location information from the DHS survey cluster locations to estimate the respondents’ proximity to the nearest health facilities. Following accepted practices, the travel time estimates described the modes of transport (hybrid walking and motorised), origin (DHS cluster location) and destination (nearest health facilities) [51]. We used travel time estimates because they account for changes in elevation and the mode of transportation.

Travel times and distance were measured to the nearest health facility because the DHS does not name health facilities. Hence, modelling proximity to the nearest health facility can be problematic as some women might bypass the nearest facility and travel longer [52]. Furthermore, the random displacement of cluster locations to preserve confidentiality could affect the travel times, but recommended distance buffers were used to moderate their effect [31]. Additionally, we did not account for the possible variations in travel times due to travel disruptions in the rainy season, road conditions, traffic congestion in urban areas and the type of transportation.

The outcome variable, “distance as a problem”, depends on the ability of the respondent to recall events rendering the response susceptible to bias. Furthermore, there could be question order bias with the importance of proximity reduced because distance to the health facility is the third question among the four assessing barriers to care. The four questions cover getting permission to see the doctor, getting money for advice or treatment, distance to the health facility and not wanting to go, in that order.

## Conclusion

This study combines different data sources to evaluate proximity to maternal health services. We joined routine health data, DHS, and travel time models to assess proximity to health services similar to other studies integrating data. The study finds a correlation between perceived and modelled proximity to health facilities. The findings unpack the inequalities in access to maternal health services and their impact. The impact of proximity is lessened when socio-demographic characteristics are improved. Hence, efforts to improve maternal health outcomes and coverage should target health facility location and the livelihood of households.

## Supplementary Information

**Additional file 1.**

**Additional file 2.**

**Additional file 3.**

## Data Availability

The DHS GMHS data that support the findings of this study are available from The DHS Program and can be downloaded at https://dhsprogram.com/data/available-datasets.cfm after due permission from the DHS program.

## Abbreviations

ANC: Antenatal Care
DHS: Demographic and Health Survey
GHS: Ghana Health Service
GMHS: Ghana Maternal Health Survey
IQR: Inter Quartile Range
PNC: Postnatal Care
SBA: Skilled Attendant/Attendance at Birth
