# Supplementary material for: Distance is “a big problem”: a geographic analysis of reported and modelled proximity to maternal health services in Ghana
Source: BMC Pregnancy Childbirth. 2022 Aug 31;22:672. doi: 10.1186/s12884-022-04998-0 (PMC9429654; doi:10.1186/s12884-022-04998-0)
Supplement: Supplementary file 1 — Additional file 1. [file 12884_2022_4998_MOESM1_ESM.docx]

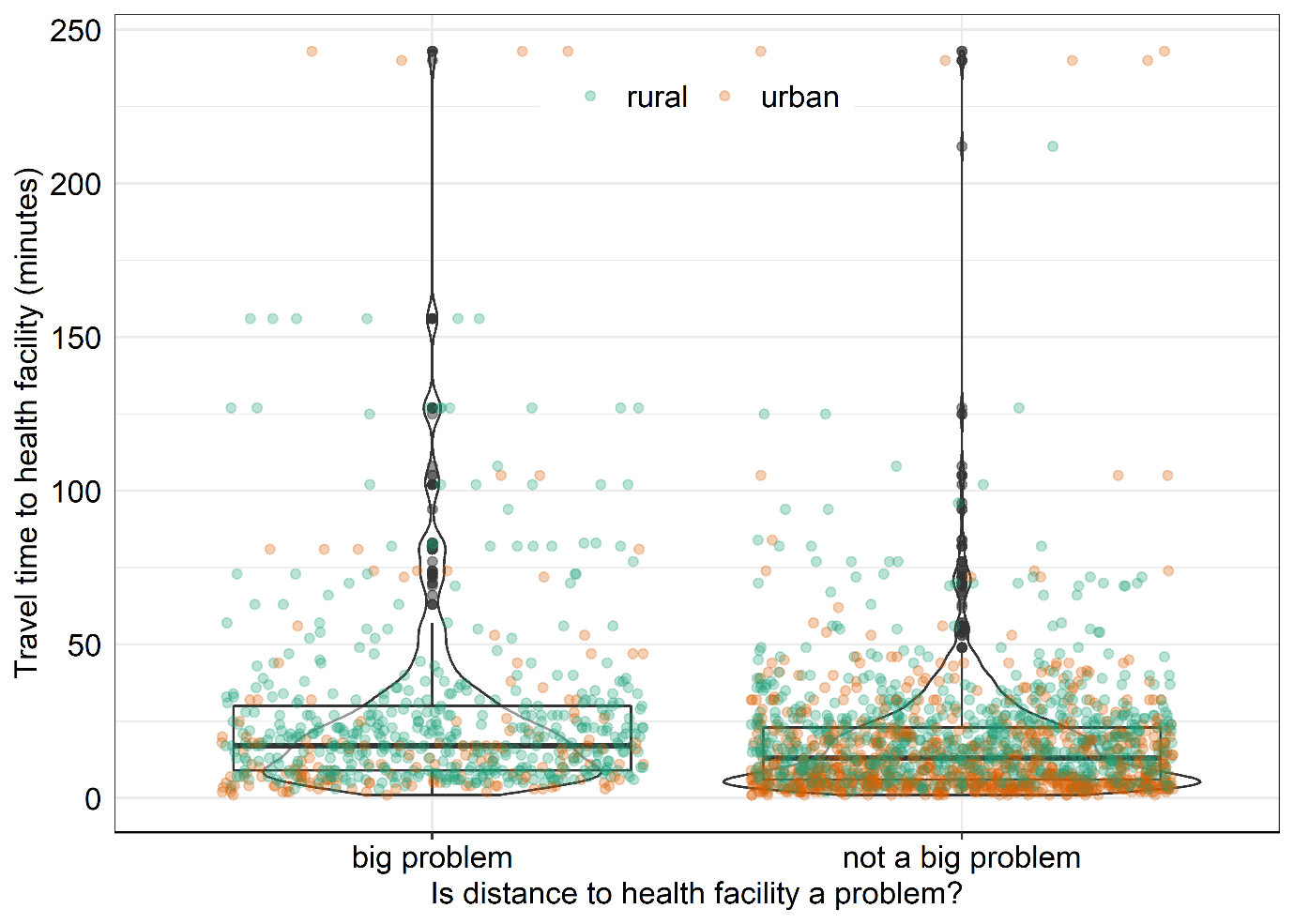


Figure S1: Modelled travel time to the nearest health facility providing birthing services versus women reporting distance as a big problem for rural and urban GMHS cluster locations. The boxplot shows the median travel times and interquartile range, the dots present the rural/urban distribution of the women and the violin plots show the density distribution of the women.
