# Supplementary material for: Distance is “a big problem”: a geographic analysis of reported and modelled proximity to maternal health services in Ghana
Source: BMC Pregnancy Childbirth. 2022 Aug 31;22:672. doi: 10.1186/s12884-022-04998-0 (PMC9429654; doi:10.1186/s12884-022-04998-0)
Supplement: Supplementary file 2 — Additional file 2. [file 12884_2022_4998_MOESM2_ESM.docx]

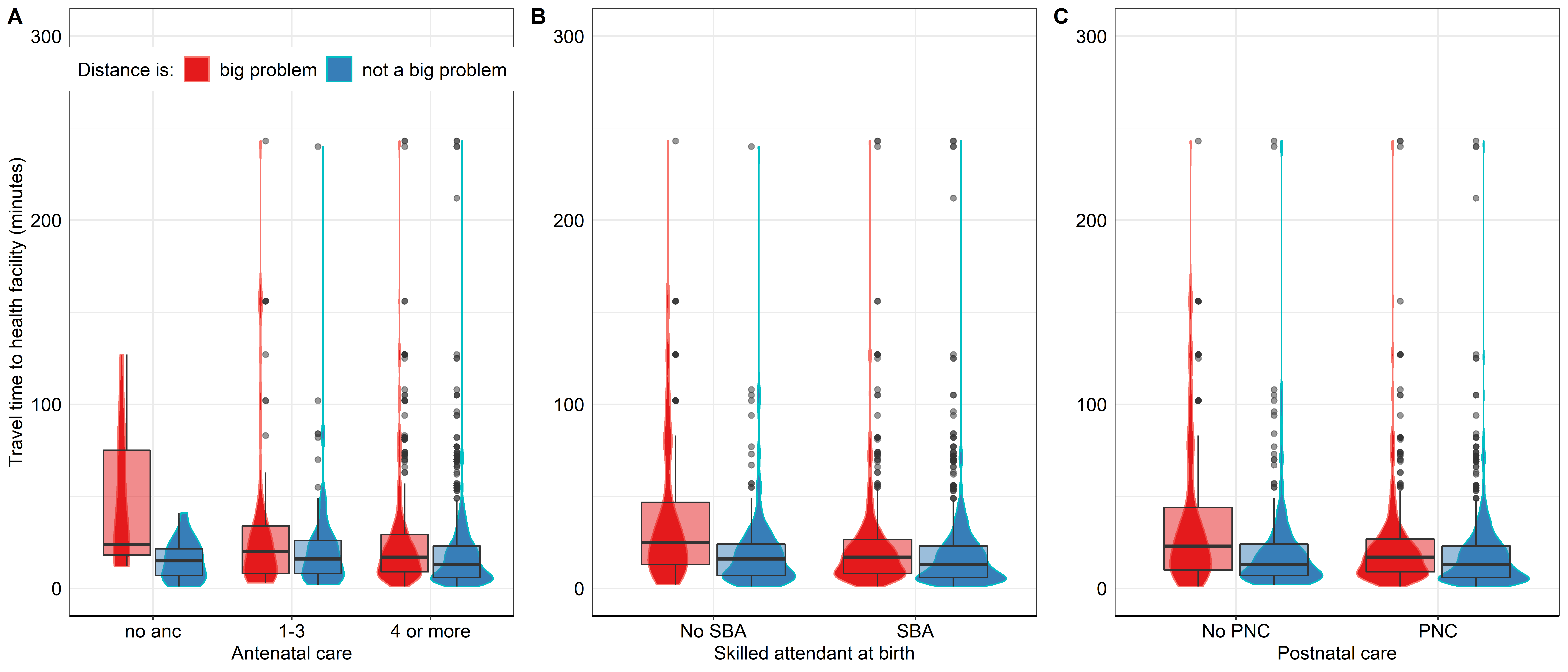


Figure S2: Modelled travel time to the nearest health facility providing birthing services versus women reporting distance as a big problem for utilisation of A. Antenatal, B. Skilled birth and C. Postnatal care services. The boxplot shows the median travel times and interquartile range and the violin plots show the density distribution of the women.
