# Supplementary material for: Distance is “a big problem”: a geographic analysis of reported and modelled proximity to maternal health services in Ghana
Source: BMC Pregnancy Childbirth. 2022 Aug 31;22:672. doi: 10.1186/s12884-022-04998-0 (PMC9429654; doi:10.1186/s12884-022-04998-0)
Supplement: Supplementary file 3 — Additional file 3. [file 12884_2022_4998_MOESM3_ESM.docx]

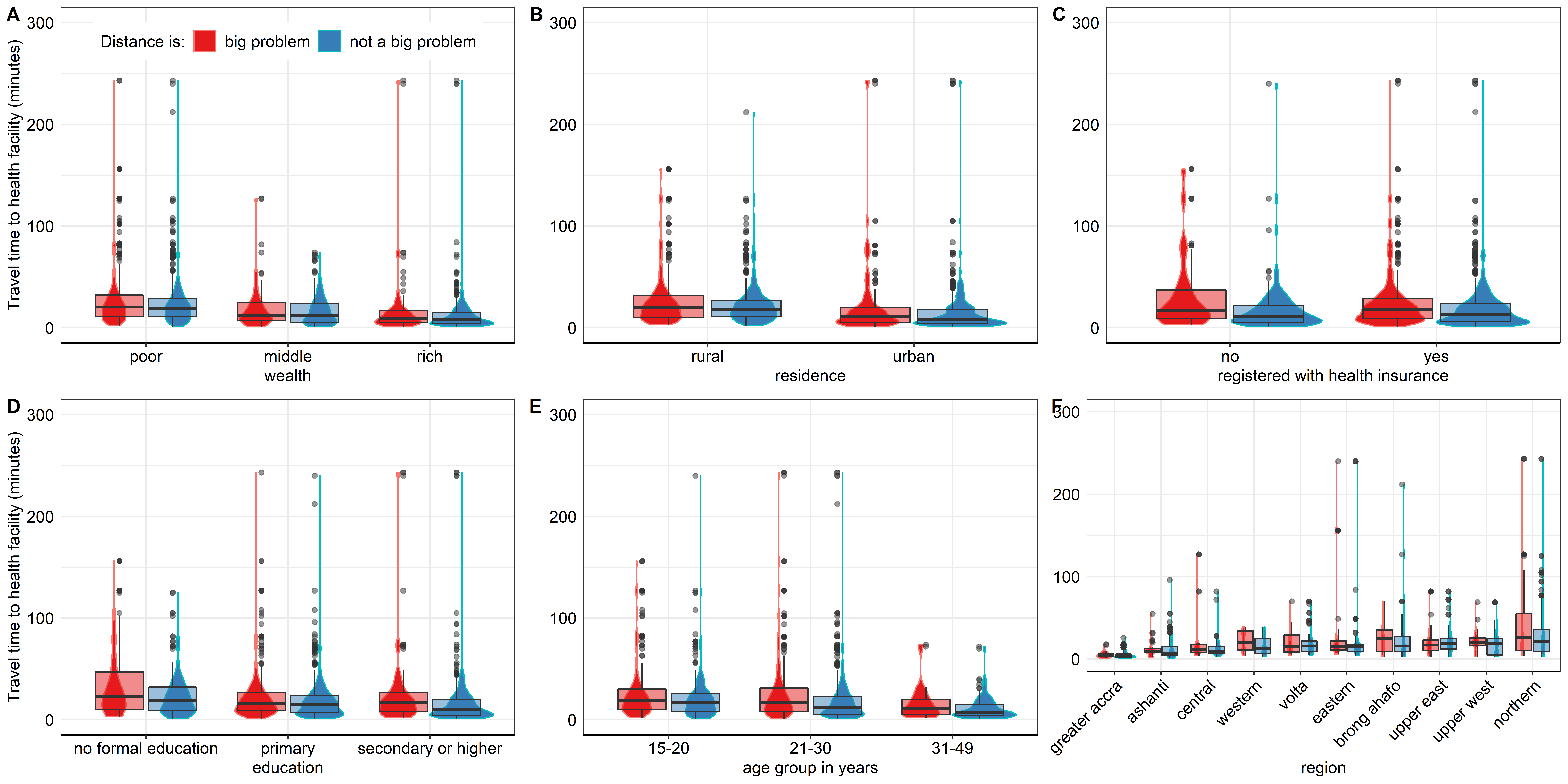


Figure S3: Travel time to the nearest health facilities providing birthing services versus reported distance as a big problem compared by A. Wealth, B. Residence C. Health insurance, D. Education, E. Age group, and F. Region. The boxplot shows the median travel times and interquartile range and the violin plots show the density distribution of the women.
